# Quantifying intrinsic chemical reactivity of molecular structural features for protein binding and reactive toxicity, using the MOSES chemoinformatics system

**DOI:** 10.1186/1758-2946-4-S1-O8

**Published:** 2012-05-01

**Authors:** Johannes AH Schwöbel, Bruno Bienfait, Johann Gasteiger, Thomas Kleinöder, Jörg Marusczyk, Oliver Sacher, Christof H Schwab, Aleksey Tarkhov, Lothar Terfloth, Mark TD Cronin

**Affiliations:** 1 Molecular Networks GmbH, Henkestraße 91, D-91052 Erlangen, Germany; 2 School of Pharmacy and Chemistry, Liverpool John Moores University, Byrom Street, Liverpool, L3 3AF, UK

## 

Covalent binding of xenobiotic compounds to endogenous biomolecular sites, e.g. protein residues, leads to potentially irreversible toxic effects such as enhanced acute toxicity or skin sensitization [1]. This mechanistic knowledge provides the basis for the *in silico* prediction of these toxicities, as required by the EU REACH legislation and the EU Cosmetics Directive. A general toxicity prediction can be based on three consecutive steps [2]: (1.) *Identification of a potential reactive protein binding mechanism via a set of molecular structural features*. Those structural features can be encoded by the Chemical Subgraph Representation Markup Language (CSRML), that supports a flexible annotation of meta information, including physicochemical properties as annotations. (2.) *Confirmation and quantification of* (*bio*)*chemical reactivity*. The potential for a chemical to be reactive can be captured by mechanistically based QSAR models. This intrinsic reactivity is calculated rapidly by descriptors of the chemoinformatics platform Molecular Structure Encoding System (MOSES) [3]. It represents electronic, steric and resonance effects in chemical structures. The performances obtained by these reactivity models are close to time-consuming quantum chemical reactivity calculations, e.g., *se* = 0.44 *versus* 0.40 for glutathione adduct formation via Michael addition, comparing predicted values to an experimental reactivity dataset [1]. (3.) *Establishing a relationship between calculated reactivity and toxicity*. The predicted intrinsic reactivity values were linked to the computational prediction for different modes of toxic action, with good correlations between predicted and *in vitro* toxicity (up to *r*^2^=0.86).

The combined use of structural information and computed reactivity could assist in the non-animal based risk assessment of chemicals for regulatory purposes and in the application of integrated testing strategies (ITS). The research has received funding from the EU FP7 COSMOS Project (grant agreement n° 266835) and financing from COLIPA.
